# The human hookworm vaccine: recent updates and prospects for success

**DOI:** 10.1017/S0022149X15000206

**Published:** 2015-04-08

**Authors:** M.E. Bottazzi

**Affiliations:** Sabin Vaccine Institute and Texas Children's Hospital Center for Vaccine Development, National School of Tropical Medicine, Baylor College of Medicine, Houston, Texas, USA

## Abstract

Approximately 440 million people globally are afflicted by hookworm disease, one of the 17 WHO-recognized neglected tropical diseases (NTDs). The iron-deficiency anaemia attributed to this disease contributes to at least 3.2 million disability-adjusted life years (DALYs) according to the Global Burden of Disease Study 2010. The current WHO-recommended control strategies rely primarily on mass drug administration or preventive chemotherapy. However, evidence is starting to accumulate confirming that preventive chemotherapy alone will not be sufficient to reduce the reinfection rates of hookworm, especially in areas of heavy transmission. The global health and research community is currently building a consensus stressing the need for the advancement of research and innovation to bridge the gaps and identify new public health interventions for diseases such as hookworm and other NTDs. This paper presents the strategies used by the Sabin Vaccine Institute Product Development Partnership (Sabin PDP) in their ongoing endeavour for the development of a human hookworm vaccine. Recent updates and the current prospects for success of an effective human hookworm vaccine, as a new technology to be linked to or combined with drug interventions, are presented.

## Overview

The World Health Organization (WHO), through their Department of Control of Neglected Tropical Diseases, is a proponent for a roadmap that utilizes an integrated approach towards the prevention, treatment and diagnosis of 17 diseases that are the most prevalent diseases amongst populations living in poverty and are present in 149 endemic countries worldwide (Abroug *et al.*, 2006; World Health Organization, 2015a). Their strategies rely on public-health interventions including mass drug administration, also known as preventive chemotherapy, innovative and intensified disease management, vector control and pesticide management, the provision of safe drinking water, basic sanitation and hygiene services, and education and zoonotic disease management (World Health Organization, 2015a).

Among the 17 WHO-recognized neglected tropical diseases, human hookworm disease, caused predominantly by infection with *Necator americanus*, has been shown to afflict approximately 440 million people globally (Hotez *et al.*, 2014; Pullan *et al.*, 2014), with the highest burdens found in Asia, followed by sub-Saharan Africa and Latin America and the Caribbean. As described in the recent Global Burden of Disease Study 2010, hookworm infection contributes to 3.2 million disability-adjusted life years (DALYs), primarily attributed to iron-deficiency anaemia (Murray *et al.*, 2012).

For hookworm infection, the current WHO-recommended control strategies (World Health Organization, 2015b) rely primarily on mass drug administration or preventive chemotherapy with a single annual tablet of either albendazole or mebendazole. However, evidence is starting to accumulate confirming that preventive chemotherapy alone will not be sufficient to reduce the reinfection rates of hookworm, especially in areas of heavy transmission. There are additional concerns about the true effectiveness of mebendazole for improving anaemia when used in a single dose (Soukhathammavong *et al.*, 2012), while single-dose albendazole has also shown variability in its effectiveness at reducing worm burdens.

## Research and innovation for public-health interventions

In response to these gaps and deficiencies, the global health and research community is currently building a consensus stressing that, for the new post-2015 Millennium Development Goals agenda, research and innovation should play a very crucial and important role (Bottazzi, 2014). The need to bridge such gaps is especially important for new public health interventions for diseases such as hookworm and other NTDs (Bottazzi, 2014; PATH, 2014). As highlighted above, for hookworm disease and for the drugs mentioned above, the cure rates seem to be quite low and, following treatment, reinfection in the treated individuals appears several months later, with little or no improvement in the intensities of infection or in anaemia (Smith & Brooker, 2010; McCarty *et al.*, 2014). In fact, a recent modelling study provides initial evidence that, for hookworm transmission, preventive chemotherapy alone would likely work only if it is linked to other public-health strategies (Lustigman & Bottazzi, 2011). Briefly, the study proposes that vaccination of school-age children and women of child-bearing age living in endemic areas would provide a cost-effective control measure complementing conventional chemotherapy. The authors also note that even a vaccine with an efficacy as low as 30% could offer a substantial economic value. Therefore, the development of an anti-hookworm vaccine could be considered as a cost-effective control measure complementing conventional chemotherapy (Lee *et al.*, 2011; Lustigman & Bottazzi, 2011).

In response to the need for continued research and development (R&D) and innovation for NTDs, the WHO recently chartered a Product Development for Vaccines Advisory Committee (PDVAC) (World Health Organization, 2014). This committee will evaluate the prospects for promising R&D tools for diseases of high global burden for which no vaccines or drugs currently exist but which have some ongoing product development activity.

PDVAC could have a transformational role working in partnership with the well-established vaccine Product Development Partnerships (PDPs) (USAID, 2009; Grace, 2010; World Health Organization, 2014) and serve as a consensus builder for vaccine product development, prioritization, protocol harmonization and suitability, and public policy for global access.

The Sabin Vaccine Institute Product Development Partnership (Sabin PDP) and its laboratories in Houston, Texas (The Sabin Vaccine Institute and Texas Children's Hospital Center for Vaccine Development), launched a programme in the year 2000 to establish and evaluate the biological feasibility for the development of a hookworm vaccine.

## The Human Hookworm Vaccine Initiative (HHVI)

Hookworm infection does not appear to induce a natural protective immune response in the human host. Instead, human hookworms are strong immunomodulators, which enable infections to persist for years and be present even in elderly populations (Bethony *et al.*, 2002). There are studies and reviews that provide the basis of helminth biology and their immune responses and mechanisms of immunomodulation (Finkelman *et al.*, 2004; Maizels *et al.*, 2004; Anthony *et al.*, 2006; Nair *et al.*, 2006; Tribolet *et al.*, 2015). The biological feasibility for vaccine development was first evaluated in the veterinary field with a canine hookworm vaccine, which was marketed in the United States in the 1970s. This vaccine relied on a radiation-attenuated infective larva stage (L3) vaccine. It achieved high levels of protection against disease due to *Ancylostoma caninum* but was later discontinued due to the high cost of production and complex storage and distribution issues (Schneider *et al.*, 2011; Hotez *et al.*, 2013).

The Human Hookworm Vaccine Initiative (HHVI) of the Sabin PDP has an overall objective to develop a human hookworm vaccine (HHV) (Hotez *et al.*, 2003, 2013). The proposed target product profile of the HHV proposes that the vaccine would: (a) be intended for at-risk children under the age of 10 years; (b) be administered by intramuscular injection; (c) include up to two doses and be stored at between 2 and 8°C; (d) be administered concurrently with other childhood vaccines, such as the measles vaccine; (e) have an efficacy of at least 80% in preventing moderate and heavy hookworm infections caused by *N. americanus* and the resulting intestinal blood loss and anaemia (Loukas *et al.*, 2005). It is estimated that more than 80% of human hookworm cases are caused by *N. americanus* (Stoll, 1947).

We propose that for the HHV the achievement of sterilizing immunity will not be required in order to deliver clinical benefit. Since the clinical pathology of human hookworm infections is based primarily on the proportional relationship between worm intensities and their ability to cause intestinal blood and protein loss, the overall goal of a vaccine would be to reduce the likelihood of developing severe hookworm infections, which will result in the reduction of blood and nutrient loss to a level that is not associated with clinical disease (Hotez *et al.*, 2013).

Vaccine target antigen discovery and selection to identify the hookworm macromolecules essential to worm survival focused on several key criteria: (a) efficacy in animal trials; (b) immuno-epidemiological observations in individuals residing in endemic areas; (c) feasibility of protein expression and scaled-up manufacture using low-cost expression systems such as yeast, bacteria or plants; and (d) a plausible mechanism of protection associated with them (Tribolet *et al.*, 2015).

The HHVI first used a parallel approach to identify and test antigens from both the L3 stage (which could reproduce the protection seen by the live attenuated vaccine) and antigens from the adult worm stage (Tribolet *et al.*, 2015). Both strategies identified recombinant antigens, with animal proof-of-concept studies showing protection against challenge infections. However, development of the leading L3-stage candidate against *N. americanus*, *Na*-ASP-2 hookworm vaccine, was halted following results from clinical trials in Brazil where a subset of chronically infected subjects with high pre-vaccination IgE titres to larval antigens experienced generalized urticaria following vaccination (Diemert *et al.*, 2012).

Better success was achieved by targeting the blood-feeding apparatus of the adult hookworm. The strategy of identifying suitable vaccine targets using gut-expressed antigens has been described previously for the gastrointestinal nematode parasites of ruminants, such as *Haemonchus contortus*, which have been shown to be protective as recombinant protein-based vaccines (Knox & Smith, 2001; Knox *et al.*, 2003; Knox, 2011). Therefore, from approximately two dozen proteins that are putatively involved in the adult hookworm blood-feeding process, the two lead candidate antigens, the aspartic protease haemoglobinase APR-1 (modified by site-directed mutagenesis to abolish the protease catalytic activity – *Na*-APR-1(M74)), and the glutathione S-transferase (*Na*-GST-1), have both shown proof-of-concept of efficacy in laboratory dogs and, in the case of *Na*-APR-1(M74), induction of neutralizing antibodies against multiple heterologous strains of the parasite (Hotez *et al.*, 2013). *Necator americanus* APR-1 is structurally and antigenically very similar to *A. caninum*-APR-1. In the studies with *Na*-APR-1(M74), the induction of neutralizing antibodies has been shown against challenges with both these strains of the parasite (Loukas *et al.*, 2005). These *Necator* antigens have now been selected for product and clinical development (Jariwala *et al.*, 2010; Goud *et al.*, 2012; Plieskatt *et al.*, 2012; Curti *et al.*, 2014).

Both *Na*-GST-1 and *Na*-APR-1(M74) vaccines have been manufactured as recombinant antigens formulated on an aluminium hydroxide adjuvant (Alhydrogel^®^). The monovalent *Na*-GST-1 and *Na*-APR-1(M74) vaccines are currently in phase 1 clinical trials in the USA and Brazil, and there are plans to advance clinical testing in Gabon, Africa. Clinical testing is also evaluating whether additional adjuvants will be required to achieve acceptable immunogenicity. Such adjuvants include synthetic Toll-like receptor (TLR) agonists, such as glucopyranosyl lipid A (GLA) or CpG oligodeoxynucleotide (Hotez *et al.*, 2010). Clinical endpoints of the HHV are being developed in parallel with parasitological endpoints, including number of worms, faecal egg counts and faecal blood loss. Neutralizing anti-enzyme antibodies are also being developed as potential surrogate correlates of immunity. Following phase 1 testing of each individual antigen in adults, they will also be evaluated for safety and immunogenicity in co-administration strategies, with the ultimate goal to develop and test the efficacy of a single co-formulated product.

The bulk of development of the HHV has been supported primarily through academic and public–private product development partnerships (Bottazzi & Brown, 2008; Hotez *et al.*, 2010, 2013; Maisonneuve *et al.*, 2014), and is also linked to a European Commission-supported Framework Program 7 (FP7) project known as HOOKVAC (HOOKVAC, 2014). Financial support has employed a variety of sources, including funding from private/philanthropic sources such as the Bill & Melinda Gates Foundation and also via strong partnerships with the governments of Brazil, The Netherlands and the European Union. New and increased financing from major funders will be critical to advance these candidate vaccines. In addition, three possible different global access and regulatory strategies have been proposed: (1) registration in an endemic country where the vaccine will be manufactured at industrial scale (e.g. Brazil or another disease-endemic country), followed by WHO prequalification; (2) article 58 of the European Agency; or (3) the US Food and Drug Administration (FDA).

## Summary

Widespread use of an effective HHV, ideally linked or combined to drug interventions, would significantly improve global public health, averting up to 3.2 million DALYs annually and greatly reducing a leading cause of global anaemia (Lustigman & Bottazzi, 2011; Murray *et al.*, 2012). As outlined above, it could also become a critical technology for the eventual elimination of hookworm infection in low- and middle-income countries. Such a vaccine has been described as an ‘antipoverty vaccine’ because of its potential to improve the economic development of affected populations (Hotez, 2011). Also, due to the additive effect of concurrent infection with malaria and hookworm on severity of anaemia, the HHV in could potentially reduce the burden of disease due to *Plasmodium falciparum* in sub-Saharan Africa (Brooker *et al.*, 2007).

## Acknowledgements

Maria Elena Bottazzi thanks Dr Peter Hotez, Dean of the National School of Tropical Medicine for his suggestions during the preparation of this paper. No writing assistance was utilized in the production of this manuscript.

## Financial support

M.E.B. receives funding from non-profit organizations to develop a human hookworm vaccine (Bill & Melinda Gates Foundation, Dutch Ministry of Foreign Affairs and European Union FP-7 Program via the Sabin Vaccine Institute).

## Conflict of interest

The author has no other relevant affiliations or financial involvement with any organization or entity with a financial interest in, or financial conflict with, the subject matter or materials discussed in this paper, apart from those disclosed.
